# *In situ* construction of multifunctional Li_2_Si_2_O_5_/LiAlSiO_4_/C networks on micron silicon anodes for high initial coulombic efficiency

**DOI:** 10.1039/d6ra02190e

**Published:** 2026-05-26

**Authors:** Jingyu Yang, Xikai Zhou, Junfeng Rong

**Affiliations:** a Sinopec Research Institute of Petroleum Processing Co., Ltd Beijing 100083 PR China Rongjf.ripp@sinopec.com

## Abstract

The commercialization of silicon anodes is impeded by their initial coulombic efficiency (ICE) and poor cycling stability. Although pre-lithiation has emerged as a promising strategy to address these challenges, existing approaches suffer from high costs, complex processing, and inadequate environmental stability of pre-lithiated products. Herein, we report a scalable solid-state synthesis strategy. Using low-cost fly ash-derived activated carbon as a multifunctional precursor and LiOH as the lithium source, an integrated micro-silicon-based composite was constructed *via in situ* solid-state reactions during high-temperature treatment. The resulting composite simultaneously incorporates Li_2_Si_2_O_5_ as a pre-lithiation agent, LiAlSiO_4_ as a fast ion conductor, and a conductive/buffering carbon network. The composite delivers a high reversible specific capacity of 1564.3 mA h g^−1^ while substantially improving the ICE of micro-silicon anodes from 77.9% to 93.3%. By optimizing the ratio of lithium source to silicon, the ICE can be further enhanced to 101.1%. Systematic phase evolution analysis and comparative experiments elucidate the synergistic mechanisms of Li_2_Si_2_O_5_-mediated pre-lithiation and LiAlSiO_4_-reduced interfacial impedance. This work not only presents a new pathway for high-value utilization of fly ash but also provides novel design principles for pre-lithiation of high-performance micro-silicon-based anodes.

## Abstract 2

The commercialization of silicon anodes is impeded by their initial coulombic efficiency (ICE) and poor cycling stability.

## Graphical abstract

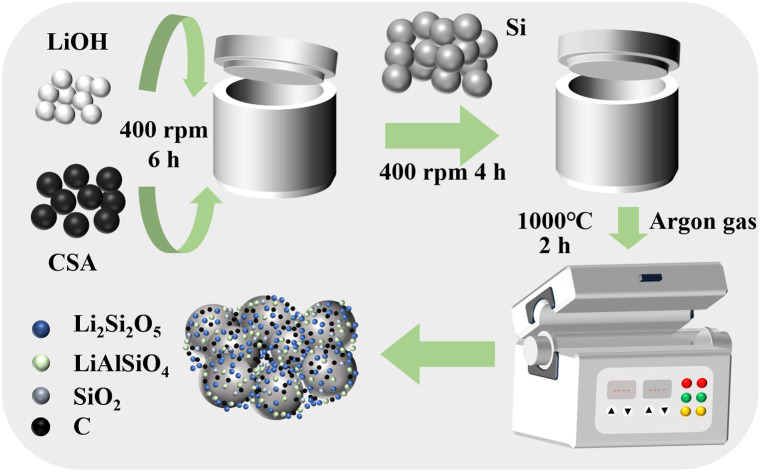

## 1. Introduction

The energy density of lithium-ion batteries (LIBs) is a key indicator determining the range of consumer electronics and electric vehicles.^1–4^ Currently, the specific capacity of commercial graphite anodes has approached its theoretical limit, making it difficult to meet the growing energy storage demands, and there is an urgent need to develop anode materials with higher specific capacity.^5,6^ Silicon (Si) is regarded as a research hotspot for next generation anode materials due to its high theoretical specific capacity (4200 mA h g^−1^), appropriate working potential, and abundant resource reserves.^7,8^ However, the practical application of Si faces multiple challenges. The alloying/dealloying process of Si is accompanied by huge volume expansion (>300%),^9^ which leads to electrode structure cracking and repeated fracture and reformation of the solid electrolyte interphase (SEI) film, causing rapid capacity decay.^10,11^ Meanwhile, its poor intrinsic electronic conductivity restricts rate performance.^12,13^ In addition, the irreversible consumption of a large amount of active lithium during the initial lithiation process results in the ICE of Si anodes being generally lower than 80%.^14–16^ Low ICE means that a large amount of lithium from the cathode is consumed during the first charge/discharge cycle, which directly reduces the energy density of full cells and becomes a key bottleneck restricting the commercial application of Si anodes.^17–20^

Pre-lithiation treatment, which introduces additional lithium sources into the material to compensate for the initial irreversible capacity loss, is an effective means to improve the ICE of Si anode materials.^21,22^ Zhao *et al.* prepared micron-scale silicon thin-film electrodes and applied electrochemical prelithiation, resulting in an increase of the half-cell ICE from approximately 70% to 89%.^23^ Although effective, this electrochemical prelithiation method requires precise potential control and specialized cell assembly, limiting its scalability.^24,25^ Peng *et al.* employed chemical prelithiation using organolithium reagents (LiFSI) on micron silicon powders, increasing the half-cell ICE from 77.9% to 92.9%.^26^ However, the use of highly reactive chemicals and flammable solvents raises safety and environmental concerns.^27–29^ Nguyen *et al.* applied a direct contact prelithiation approach, in which stabilized lithium metal powder (SLMP) was uniformly deposited onto micron Si composite electrodes *via* a spray coating method, resulting in a half-cell ICE improvement from approximately 79% to 95%.^30^ Nevertheless, the high cost of SLMP and the challenge of achieving uniform lithium distribution remain limitations.^31–33^ Thermal solid-state prelithiation, valued for its operational simplicity, has also been extensively studied. Jeong *et al.* synthesized Si/Li_*x*_SiO_*y*_ composite anodes by thermal reduction using metallic lithium, enhancing the half-cell ICE of micron silicon to 83.5%.^34^ Zhu *et al.* prepared Si/Li_2_SiO_3_ nanocomposites using nanosilicon and performed thermal solid-state prelithiation with LiBH_4_, achieving a half-cell ICE of approximately 89% while improving mechanical stability through heterogeneous microstructures that buffer volume expansion.^35^ Similarly, Kamma *et al.* employed LiH to prelithiate micron silicon, forming Li_*x*_Si; the corresponding micron Si/graphite half-cells achieved a maximum ICE of 118.3%, with 54.9% capacity retention after 100 cycles.^32^ Although these prelithiation techniques can significantly enhance the ICE of silicon-based anodes, they are generally limited by (i) reliance on highly reactive lithium sources or complex synthesis conditions, (ii) insufficient environmental stability of the prelithiated products, and (iii) a focus on either SEI modification or alloy formation without integrating ionic conduction pathways.

In silicon-based anodes, pre-lithiation aims to generate various lithium silicates, including Li_2_Si_2_O_5_, Li_2_SiO_3_, Li_4_SiO_4_, and Li_6_Si_2_O_7_.^28,35^ The low-lithium phases Li_2_Si_2_O_5_ and Li_2_SiO_3_ are electrochemically active and can release lithium ions during charge–discharge,^36,37^ compensating for the initial irreversible lithium loss. In contrast, the high-lithium phases Li_4_SiO_4_ and Li_6_Si_2_O_7_ are electrochemically inert,^38,39^ serving primarily to suppress the volume expansion of silicon. Further pre-lithiation leads to the formation of Li_*x*_Si within the silicon matrix.^40^ Compared with highly reactive Li_*x*_Si, lithium silicates exhibit strong lithium storage capacity and lower reactivity, contributing to higher initial coulombic efficiency and enhanced structural stability. Additionally, LiAlSiO_4_ functions as a beneficial phase for interfacial lithium-ion transport.^41,42^ Compared with conventional ion conductors such as positive thermal expansion LLZO,^43^ electrochemically inert Al_2_O_3_,^44^ or low-conductivity negative thermal expansion ZrW_2_O_8_,^45^ LiAlSiO_4_ demonstrates a unique thermomechanical–ionic synergistic effect: it exhibits high room-temperature ionic conductivity (1.2 × 10^−7^ S cm^−1^) and negative thermal expansion (−6.2 × 10^−6^ K^−1^) over 25–1000 °C,^46,47^ enabling active contraction during battery heating, which buffers silicon expansion, reduces stress, and enhances thermal safety. Its combination of high ionic conductivity, negative thermal expansion, and low cost provides unique advantages in improving the lithium-ion kinetics, structural integrity, and cycling stability of silicon anodes.^41^ Therefore, developing a low-cost, water-stable pre-lithiation strategy that can synergistically construct Li_*x*_SiO_*y*_/LiAlSiO_4_ composite interfaces holds significant scientific value and practical potential for high-performance silicon anodes.

Herein, we report a facile *in situ* solid-state reaction strategy. Using fly ash-derived activated carbon (CSA) rich in SiO_2_, Al_*x*_SiO_*y*_, and carbon as a multifunctional precursor, and LiOH as the lithium source, a spatially coupled multifunctional interface is constructed on micron-sized silicon through ball milling and subsequent high-temperature treatment. During this process, Li_2_Si_2_O_5_, acting as a pre-lithiation agent, and LiAlSiO_4_, serving as a fast ion conductor, are generated *in situ* around Si particles, while the intrinsic carbon component in CSA forms a conductive and buffering network. Different from conventional pre-lithiation strategies that mainly rely on a single lithium source or a single protective component, this method integrates lithium compensation, interfacial Li^+^ transport, electronic conduction, and structural buffering within one interface. The resulting composite delivers an initial coulombic efficiency of up to 93.3% and exhibits excellent air and aqueous stability. This study further reveals the phase evolution and synergistic mechanism of the composite, providing insight into the design of high-performance micron-Si-based anodes with high ICE and improved cycling stability.

## 2. Experimental

### 2.1 Preparation of CSALi

CSA and LiOH were mixed at a mass ratio of 3 : 1 and placed in ball-milling jar. The mixture was then ball-milled at 400 rpm for 6 h. The resultant sample was named CSALi.

The CSALi powder was heated in an argon atmosphere to preset temperatures of 600, 700, 800, 900, and 1000 °C, respectively, at a rate of 5 °C min^−1^ and held for 2 h. The resultant samples were named CSALi-*T* (*T* = 600, 700, 800, 900, 1000).

### 2.2 Preparation of CLS

CSALi and micron-Si were mixed at different mass ratios (1 : 0.5, 1 : 1, 1 : 1.5, 1 : 2, 1 : 2.5, and 1 : 3) and ball-milled at 400 rpm for 4 h. The aforementioned samples were heated to 1000 °C under the same conditions (heating rate and atmosphere) and maintained for 2 h. The resultant samples were named CLS105-1000, CLS11-1000, CLS115-1000, CLS12-1000, CLS125-1000, and CLS13-1000, respectively.

### 2.3 Preparation of CLS12-*T*

The CSALi and micron-Si powders were mixed at mass ratio of 1 : 2 and ball-milled at 400 rpm for 4 h, and then heated in an argon-shielded tube furnace to predefined temperatures (600, 700, 800, and 900 °C) under the same conditions (heating rate of 5 °C min^−1^ and argon atmosphere) for 2 h. The resultant samples were named CLS12-*T*.

### 2.4 Preparation of comparative samples

All samples were calcined at 1000 °C under argon atmosphere for 2 h after preparation. Ball milling conditions (1) and (2) were identical to those described in Sections 2.1 and 2.2, respectively:

(1) Si: micron-Si subjected to ball milling condition (2) only.

(2) CSASi: CSA and micron-Si mixed at a mass ratio of 1 : 2, and ball milled under condition (2).

(3) LiOHSi: LiOH and micron-Si mixed at a mass ratio of 1 : 2, and ball milled under condition (1).

(4) SOLS: SiO_2_ and LiOH were pre-mixed at a mass ratio of 1 : 1 and ball-milled under condition (1). Subsequently, the SiO_2_/LiOH mixture and micron-Si were then mixed at a mass ratio of 1 : 2 and ball-milled under condition (2).

(5) CSCSOLS: the SiO_2_/LiOH mixture from sample (4) was combined with 12 wt% coconut shell-derived activated carbon and ball milled under condition (1). The resulting CSC–SiO_2_/LiOH composite was then mixed with micron-Si at a mass ratio of 1 : 2 and ball-milled under condition (2).

(6) CSCLS: coconut shell-derived activated carbon and LiOH were mixed at a mass ratio of 0.14 : 1 and ball-milled under condition (1). The resulting CSC–LiOH composite was then mixed with micron-Si at a mass ratio of 1 : 2 and ball-milled under condition (2).

### 2.5 Material characterizations

The phase composition and unit cell parameters of the sample materials were analyzed using the X-ray powder diffraction instrument (XRD) from the American Company Philips. The test employed a Cu target Kα light source, with a working voltage of 40 kV, a wavelength of *λ* = 0.154 nm, a scanning speed of 5° min^−1^, a scanning range of 5°–70°, and a step size of 0.02°. The morphology and elemental distribution of the sample materials were characterized using the FEI Quanta 400 scanning electron microscope (FE-SEM) and the attached energy dispersive spectrometer (EDS), with an acceleration voltage of 20 kV. The morphology and microstructure of the sample materials were observed using the Hitachi H-800 transmission electron microscope (HR-TEM) produced by Japan's Hitachi Corporation. The elemental valence states and relative contents on the surface of the sample materials were determined by using the Thermo Fisher Thermo ESCALAB 250 X-ray photoelectron spectroscopy instrument (XPS). The Al Kα X-ray emission source was employed, with a power of 300 W. The C 1s (284.8 eV) peak was used as the reference peak for charge correction. The local structure of the sample material was determined using the LabRAM HR UV-NIR type laser confocal Raman spectrometer (Raman) produced by HORIBA Co., Ltd of Japan. The excitation light source wavelength was 532 nm He–Ne laser, with a 50× objective lens, a confocal vacuum diameter of 100 µm, and the measurement was repeated twice to obtain the average value. The Mettler thermogravimetric analyzer TGA2 was used to monitor the changes in the mass and reaction heat of the sample materials with temperature. The test atmosphere was air, and the temperature range was 25–1000 °C with a heating rate of 5 °C min^−1^.

### 2.6 Electrochemical characterizations

CLS, Super-P, CMC, and SBR were mixed at a mass ratio of 91 : 4 : 2 : 3 in a vacuum mixer. The resulting slurry was coated onto copper foil using a 100 µm doctor blade and dried at 100 °C for 12 h in a vacuum oven. The dried electrode was roll-pressed and punched into 12 mm diameter disks (active mass: 2.5–3.5 mg), which were weighed and stored in a glove box. CR2032 coin cells were assembled in an Ar-filled glove box using the prepared electrode as the working electrode, Li metal as the counter electrode, 1 mol L^−1^ LiPF_6_ in EC : DMC : DEC (1 : 1 : 1 by volume) as the electrolyte, and Celgard 2300 as the separator. Galvanostatic charge/discharge tests were performed using a LAND CT3001A system at 25 °C within a voltage window of 0.005–1.5 V. The first three cycles were conducted at 0.1C (based on a theoretical capacity of 1500 mA h g^−1^). All key electrochemical tests were repeated in at least three independently assembled cells, and the data presented in the text are representative results.

## 3. Results and discussion

### 3.1 Characterization of CSA

**Fig. 1:**
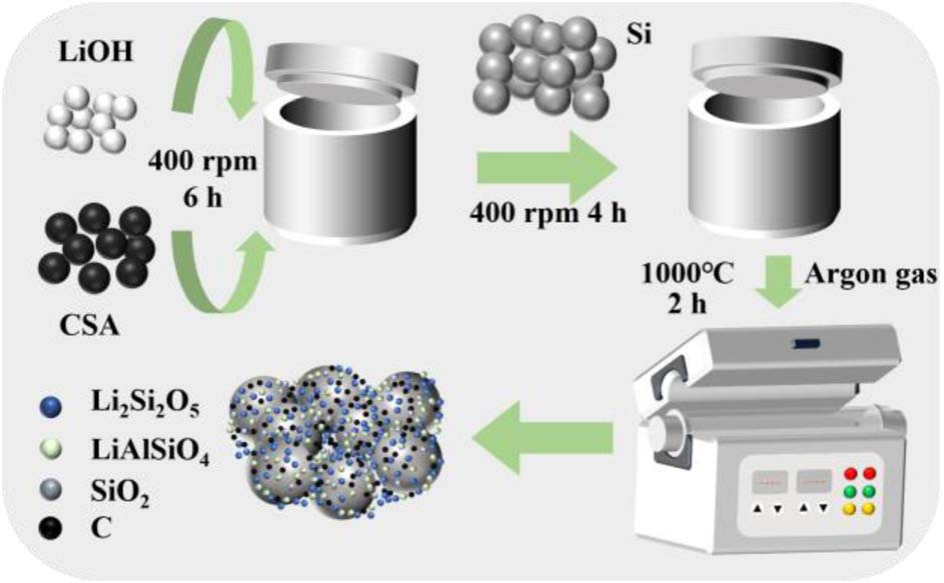
Schematic illustration of preparation process.

A facile method for pre-lithiation of micron-silicon is shown in Fig. 1. Through two steps of ball milling, the micron-silicon, LiOH and fly ash-based activated carbon are mixed. A solid-phase reaction is triggered at high temperature to *in situ* construct the Li_2_Si_2_O_5_/LiAlSiO_4_/C network on the surface of the micron-silicon.

**Fig. 2:**
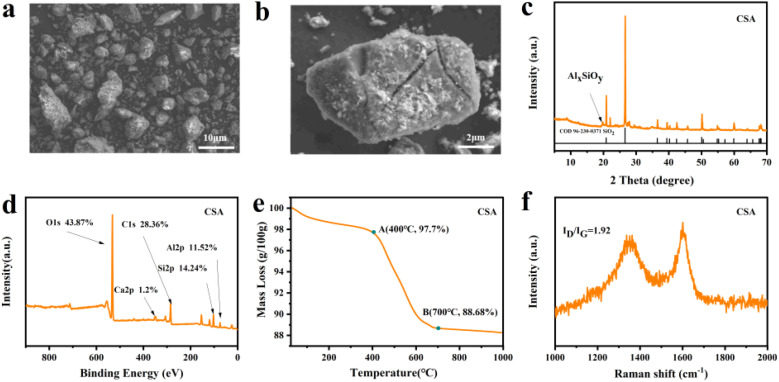
Characterization of CSA (a and b) SEM images, (c) XRD pattern, (d) XPS survey spectrum, (e) TG curve, (f) Raman spectrum.

The CSA material was characterized to elucidate its intrinsic properties and potential roles in the subsequent composite design. SEM images (Fig. 2a and b) reveal irregular polyhedral morphology of CSA with particle sizes ranging from several to tens of micrometers. The particle surfaces feature rough topography with localized cracks and fissures. EDS mapping (Fig. S1) demonstrates substantial overlap in the distribution of Si, O, and Al elements, suggesting the presence of Si–O, Al–O, and Si–O–Al bonds. XRD analysis (Fig. 2c) further identifies SiO_2_ and Al_*x*_SiO_*y*_ as the primary phases,^48^ which can serve as reactants for subsequent composite synthesis.

XPS analysis quantified the composition of CSA, which contains C (28.36%), O (43.87%), Si (14.24%), Al (11.52%), and trace amounts of Ca (1.19%) (Fig. 2d). Thermogravimetric analysis in air revealed a mass loss of 12% upon heating to 1000 °C (Fig. 2e), corresponding to a carbon content of about 10%. The residual weight of 88% at 1000 °C confirms that the primary component of CSA is an inorganic mineral matrix composed of SiO_2_ and Al_*x*_SiO_*y*_. The Raman spectrum (Fig. 2f) showed that the *I*_D_/*I*_G_ ratio was 1.92, indicating the carbon component is mainly amorphous. Due to its high ash content, CSA lacks the well-developed microporous structure characteristic of conventional activated carbons, exhibits a BET specific surface area of only 24.6 m^2^ g^−1^ (Fig. S2) and a pore size distribution predominantly in the mesoporous range (Fig. S3). This unique compositional feature of the fly ash-derived activated carbon renders it particularly suitable for subsequent solid-phase reactions by providing an adequate supply of reactants.

### 3.2 Structural characterization of composites

**Fig. 3:**
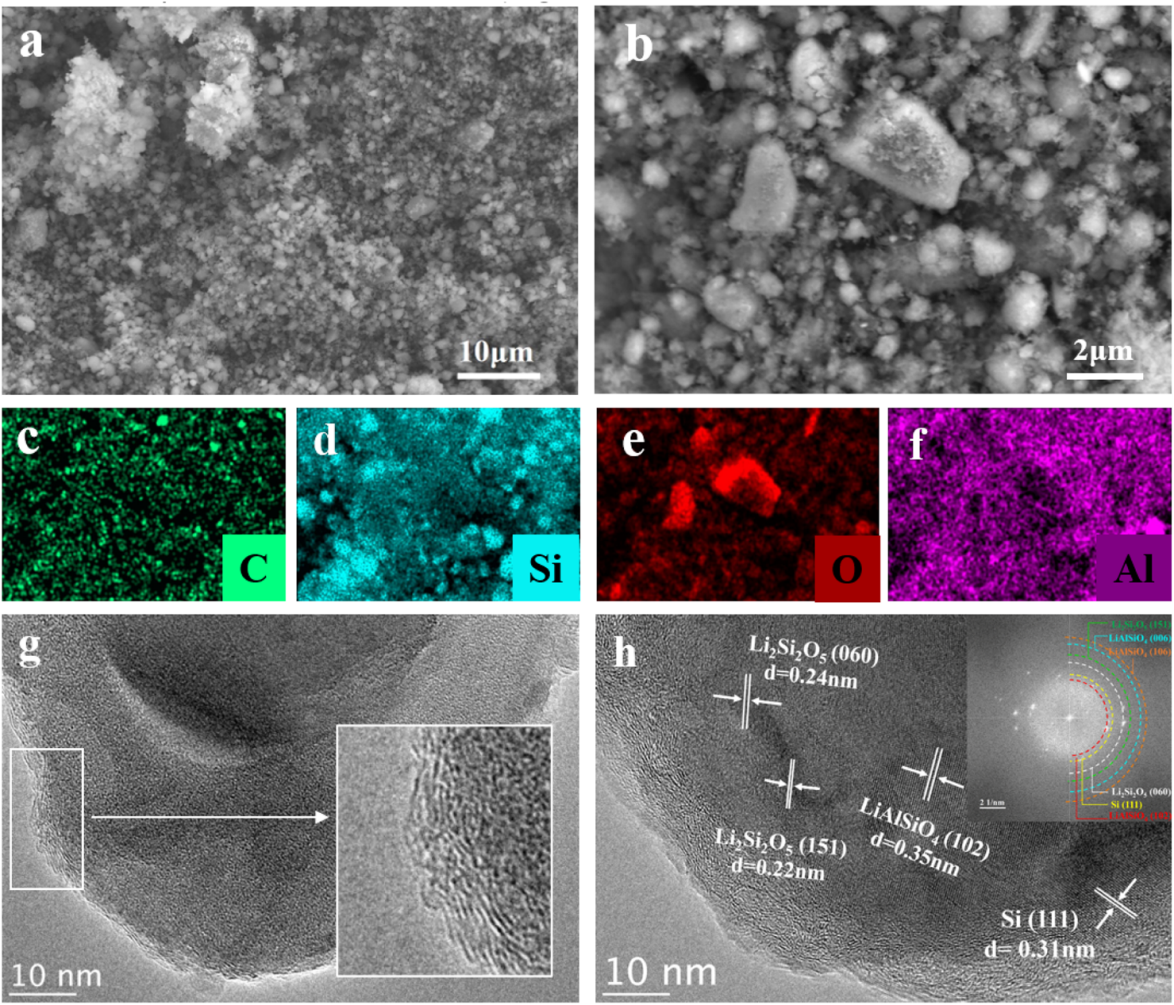
Morphology and structure of CLS12-1000. (a and b) SEM images, (c–f) EDS elemental mapping of CLS12-1000, C (c), Si (d), O (e), and Al (f), (g and h) TEM and HRTEM images and corresponding FFT patterns.

After high-energy ball-milling, the CLS12-1000 sample consists of uniformly mixed particles with irregular shapes and a narrow size distribution of 1–2 µm, as shown in the SEM images (Fig. 3a and b). The EDS elemental mapping (Fig. 3c–f) indicates that Si forms the dominant phase, while C, O, and Al are homogeneously dispersed throughout the composite, confirming effective mixing and the absence of agglomeration after ball milling and high-temperature treatment. TEM images reveal short-range ordered curved carbon fringes along the particle edges embedded in an amorphous carbon matrix (Fig. 3g), which can serve as a conductive network and a mechanical buffer to accommodate silicon expansion during lithiation. HRTEM (Fig. 3h) shows distinct lattice fringes corresponding to Si (111, *d* = 0.31 nm), Li_2_Si_2_O_5_ ((060) and (151), *d* = 0.24 and 0.22 nm), and LiAlSiO_4_ ((102), *d* = 0.35 nm). The corresponding FFT pattern further supports the coexistence of these crystalline domains in the local region. Combined with the XRD phase identification and XPS chemical-state analysis, these TEM/FFT results confirm that Li_2_Si_2_O_5_ and LiAlSiO_4_ are successfully formed and spatially coupled with Si in the CLS12-1000 composite.

**Fig. 4:**
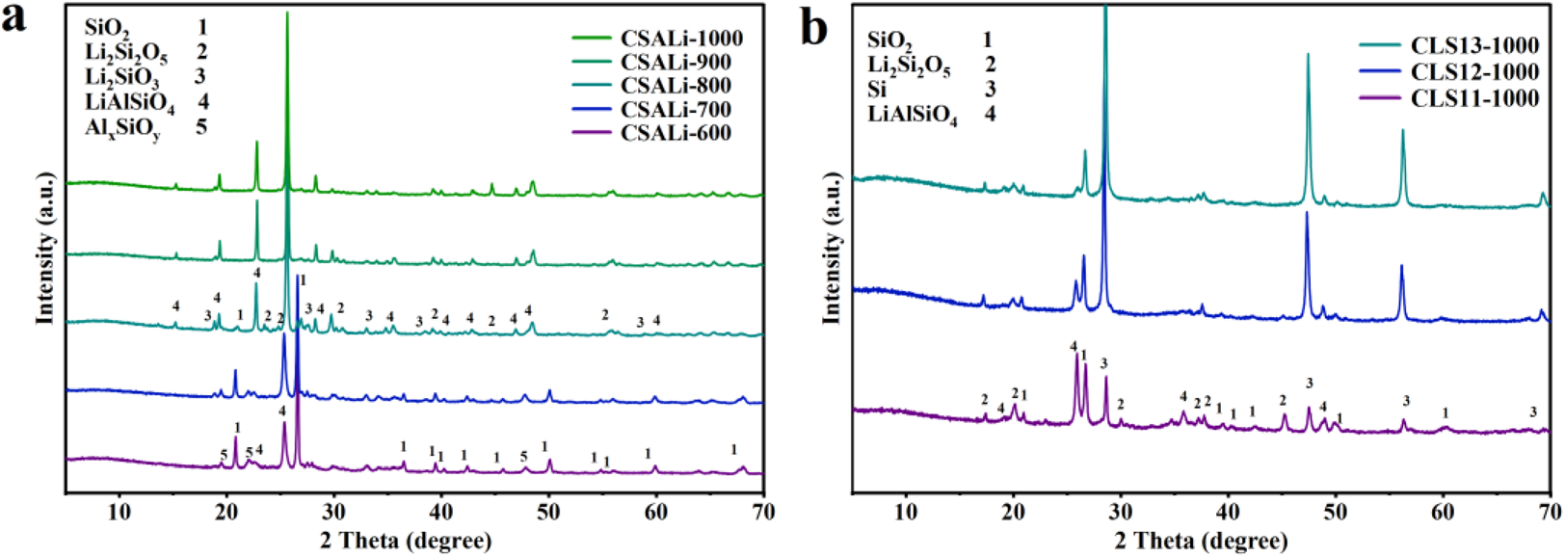
(a) XRD patterns of CSALi-*T*, (b) XRD patterns of CLS11-1000, CLS12-1000, and CLS13-1000.

Fig. 4a presents the XRD spectra of the CSALi precursor after being subjected to different temperatures (600–1000 °C) of thermal treatment, in order to investigate the high temperature solid-phase reaction laws of SiO_2_ and Al_*x*_SiO_*y*_ in CSA with LiOH. The characteristic peaks of Al_*x*_SiO_*y*_ located at 19.8° and 22.0° gradually weakened with the increase of calcination temperature (600–700 °C), and almost disappeared at 800 °C, indicating that the Al_*x*_SiO_*y*_ was continuously consumed during the high temperature process. Similarly, the SiO_2_ diffraction peak at 26.6° shows continuous intensity attenuation with increasing heat treatment temperature (600–800 °C) and completely disappears at 900–1000 °C,^49^ further confirming the continuous consumption of SiO_2_ in CSA during high temperature processing. When the temperature was 600 °C, the diffraction peak of LiAlSiO_4_ began to appear around 2*θ* = 25.4°,^41^ and the peak intensity increased with the rise in temperature. The diffraction peaks of Li_2_Si_2_O_5_ and Li_2_SiO_3_ began to appear at 600 °C, and the peak intensity continued to increase with the rise in temperature, indicating that high temperature conditions are conducive to the formation of the lithium silicate phase.^33^ As the amount of LiOH added increases, the intensity of the diffraction peaks of the lithium-containing phase continuously strengthens, which deepens the reaction degree. At the same time, electrochemically inert Li_4_SiO_4_ is also generated (Fig. S4). Therefore, in order to obtain a pure target multiphase composite material, the introduction amount of LiOH needs to be precisely controlled to avoid the complication of components caused by excessive addition. This process reveals that, upon thermal activation, LiOH undergoes localized *in situ* solid-state reactions with SiO_2_ and Al_*x*_SiO_*y*_ in CSA, gradually converting them into functional lithium silicates and LiAlSiO_4_ ion conductors. These species serve as the essential building blocks for constructing a composite interface on the silicon anode.

Fig. 4b presents the XRD patterns of CLS samples obtained by heat-treating CSALi/micro-silicon composites with different mass ratios at 1000 °C. The composites with varying mass ratios exhibit essentially identical peak shapes, with only the diffraction peak intensities differing due to the different amounts of micro-silicon added. Characteristic diffraction peaks of Li_2_Si_2_O_5_ and LiAlSiO_4_ are observable in all samples, indicating that these beneficial functional phases can still be generated when materials are composite-processed followed by calcination. The characteristic peak belonging to the Si(111) plane of the newly added micron-silicon is detected at 2*θ* = 28.4°. Differing from previous observations, Li_2_SiO_3_ diffraction peaks were not detected in any of the three samples, and simultaneously, SiO_2_ was not completely consumed after micron-silicon addition. This phenomenon may be attributed to the introduction of micron-silicon altering the reaction equilibrium, allowing partial SiO_2_ retention while simultaneously generating the thermodynamically more stable Li_2_Si_2_O_5_ phase with lower lithium-to-silicon ratio.^50^ These results indicate that, in the presence of micron-Si, the reaction products are preferentially generated *in situ* on or near the surface of Si particles rather than being randomly distributed. During ball milling, LiOH, CSA, and micron-Si are brought into close contact. Upon subsequent heat treatment, LiOH reacts with SiO_2_ in CSA to form lithium silicate species, mainly Li_2_Si_2_O_5_ under the optimized conditions, while the aluminosilicate components react with LiOH to generate LiAlSiO_4_. The gradual weakening of SiO_2_ and Al_*x*_SiO_*y*_ diffraction peaks, together with the appearance of Li_2_Si_2_O_5_ and LiAlSiO_4_ peaks in the temperature-dependent XRD patterns, supports this reaction pathway. Meanwhile, the carbon component inherited from CSA remains in the composite and forms a conductive/buffering network after high-temperature treatment. Therefore, Li_2_Si_2_O_5_, LiAlSiO_4_, and carbon are spatially coupled around micron-Si rather than simply mixed as isolated phases, enabling lithium compensation, interfacial Li^+^ transport, electronic conduction, and volume-buffering functions within one integrated interface.

**Fig. 5:**
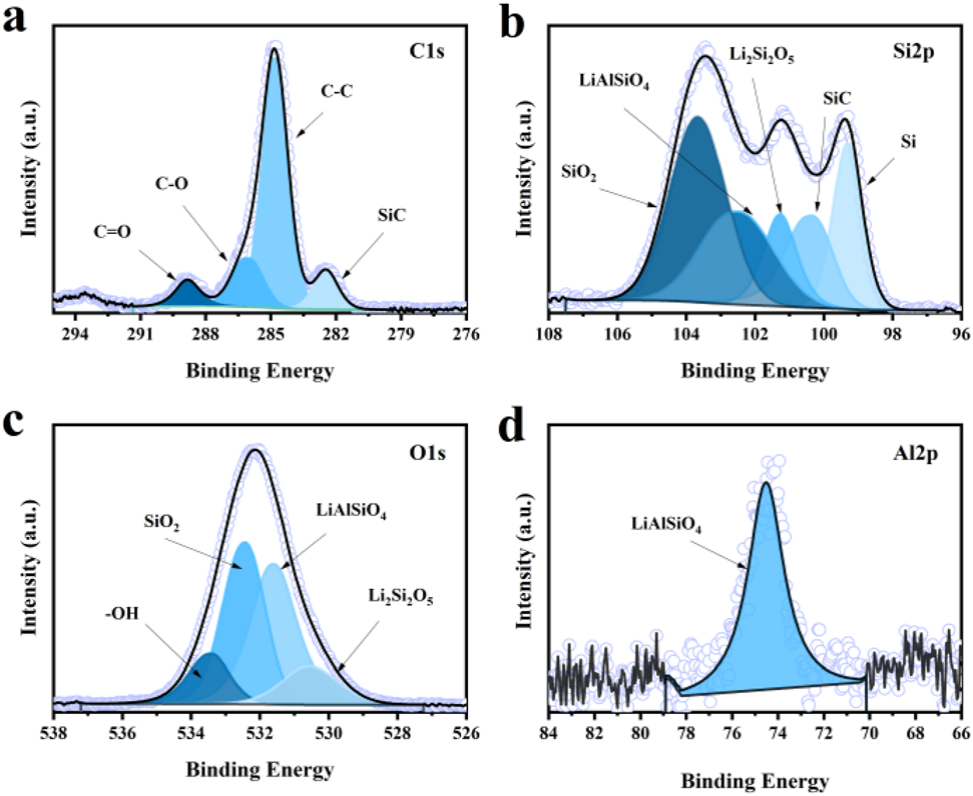
XPS spectra of CLS12-1000 (a) C 1s, (b) Si 2p, (c) O 1s, (d) Al 2p.

XPS spectroscopy of the CLS12-1000 sample confirms the presence of C, Si, O, and Al elements. In the C 1s spectrum (Fig. 5a), the main peak at binding energy of 284.8 eV is attributed to C–C bonds, while peaks at 286.1 eV and 288.8 eV correspond to C–O and C=O bonds, respectively. A characteristic peak belonging to SiC appears at 282.5 eV,^51^ however, no crystalline SiC diffraction peaks were observed in the XRD patterns (Fig. 4a and b), indicating that partial micron-silicon reacted with activated carbon to form amorphous SiC under high temperature conditions. In the Si 2p spectrum (Fig. 5b), the peak at 99.3 eV is attributed to elemental Si. The peak at 100.3 eV corresponds to SiC, the peak at 101.3 eV corresponds to Li_2_Si_2_O_5_,^52^ the peak at 102.5 eV is attributed to LiAlSiO_4_,^53^ and the peak at 103.7 eV corresponds to unreacted SiO_2_, consistent with XRD results. In the O 1s spectrum (Fig. 5c), the peak at 530.5 eV is attributed to Li_2_Si_2_O_5_, the peak at 531.6 eV corresponds to LiAlSiO_4_, the peak at 532.5 eV is attributed to SiO_2_, and the peak at 533.4 eV originates from oxygen in surface-adsorbed water or hydroxyl groups. In the Al 2p spectrum (Fig. 5d), the peak at 74.5 eV can be assigned to the Al chemical environment in LiAlSiO_4_, and the Li 1s in Fig. S5. Based on the combined XRD, XPS and TEM data, it is evident that two electrochemically beneficial phases, Li_2_Si_2_O_5_ and LiAlSiO_4_, have been successfully synthesized around micron-sized silicon *via* a high-temperature solid-state reaction. Li_2_Si_2_O_5_ provides a controllable pre-lithiation lithium source, while LiAlSiO_4_ enhances the interfacial Li^+^ transport rate. Meanwhile, the continuous conductive and buffering network formed by the carbon phase not only ensures electronic pathways but also accommodates volume expansion-induced stress. These three components create an *in situ* spatially coupled, multifunctional composite interface on the silicon surface, achieving synergistic effects of lithium compensation, enhanced ion transport, and structural buffering, thereby effectively improving the initial coulombic efficiency and cycling stability of the silicon anode.

### 3.3 Electrochemical measurements

**Fig. 6:**
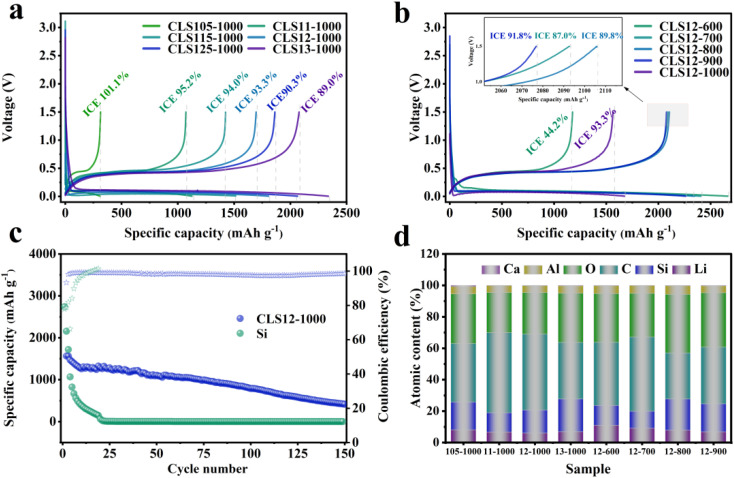
(a) Initial charge–discharge curves of CSALi/Si samples with different mass ratios calcined at 1000 °C, (b) initial charge–discharge curves of CLS12-*T* samples, (c) the cycling performance and the CE of CLS12-1000 and Si at 1C rate, (d) XPS-derived atomic composition of surface elements (Li, Si, C, O, Al, Ca) for CLS series samples.

The electrochemical performance of electrodes with different CSALi-to-Si mass ratios, prepared at 1000 °C, was evaluated by initial charge–discharge measurements (Fig. 6a). At a low silicon content (CLS105), the electrode exhibits a high ICE of 101.1%, indicating that Li_2_Si_2_O_5_ can effectively compensate for the initial irreversible lithium loss. However, the reversible capacity is limited to 313.7 mA h g^−1^, which is insufficient for practical applications. This limitation originates from the fact that Li_2_Si_2_O_5_, acting as an intermediate phase during structural evolution, partially transforms into Li_2_SiO_3_ and Si during lithiation,^54^ while Li_2_SiO_3_ possesses a relatively low reversible capacity (200 mA h g^−1^),^55^ thereby restricting the overall lithium storage capability.

With increasing silicon content, the CLS12 sample (CSALi : Si = 1 : 2) exhibits a more balanced electrochemical performance, delivering initial discharge/charge capacities of 1676.5/1564.3 mA h g^−1^ and an ICE of 93.3%. Moreover, it shows significantly improved cycling stability compared to pristine micron-sized silicon, retaining 69.5% and 26.5% of its capacity after 50 and 150 cycles at 1C, respectively (Fig. 6c), whereas the pure silicon electrode suffers rapid capacity decay to below 10 mA h g^−1^ after only 23 cycles under the same conditions. This enhancement is attributed to the synergistic construction of a multifunctional interface composed of Li_2_Si_2_O_5_, LiAlSiO_4_, and a conductive carbon network. Further increasing the silicon fraction (CLS125 and CLS13) leads to higher initial discharge capacities (1862.5 and 2077.9 mA h g^−1^, respectively), but lower ICE values (90.3% and 89.0%), indicating insufficient pre-lithiation relative to the increased amount of active silicon, resulting in incomplete surface coverage. XPS analysis (Fig. 6d) further reveals the correlation between surface composition and electrochemical performance. The XPS-derived atomic percentages provide semi-quantitative information on the surface composition of the CLS samples. As the silicon content increases from CLS105 to CLS11 and CLS12, the surface Li atomic percentage decreases from 8.12% to 6.72% and 6.02%, respectively, which is generally consistent with the decrease in ICE. This correlation indicates that the relative amount of lithium-containing surface species is closely related to the lithium-compensation capability of the electrode. However, for CLS13, the surface Li atomic percentage increases to 6.89%, while the ICE continues to decrease. This result suggests that ICE is not determined solely by the total surface Li content, but is also affected by the chemical state and spatial distribution of lithium-containing species. At an optimized CSALi-to-Si ratio of 1 : 2, LiOH can react more uniformly with SiO_2_ and Al_*x*_SiO_*y*_, leading to the formation of a well-distributed Li_2_Si_2_O_5_/LiAlSiO_4_ functional network. In contrast, excessive Si content may lead to insufficient or inhomogeneous functional coverage, which weakens the effective pre-lithiation and interfacial regulation. Therefore, a properly distributed Li_2_Si_2_O_5_/LiAlSiO_4_ network is more important than simply increasing the lithium content.

The calcination temperature also plays a crucial role in determining the electrochemical performance of CLS12 samples (Fig. 6b). The CLS12-600 sample delivers a high initial discharge capacity of 2665.1 mA h g^−1^ but a low ICE of only 44.2%. XRD analysis reveals that a large amount of SiO_2_ remains unreacted at 600 °C, and the formation of beneficial Li_2_Si_2_O_5_ and LiAlSiO_4_ phases is insufficient. During the first lithiation, the residual SiO_2_ undergoes extensive irreversible conversion into electrochemically inactive Li_4_SiO_4_,^56^ leading to severe lithium loss. With increasing calcination temperature, SiO_2_ is progressively consumed, and Li_2_Si_2_O_5_ and LiAlSiO_4_ are more fully formed, resulting in a more continuous and uniform coverage on the silicon surface. Consequently, the composite achieves the highest ICE (93.3%) at 1000 °C. XPS results (Fig. 6d) show that the surface Li atomic percentage decreases from 10.96% to 6.02% with increasing calcination temperature. This trend indicates that higher temperatures promote the solid-state reaction between LiOH and SiO_2_/Al_*x*_SiO_*y*_, transforming lithium species from a surface-enriched state into more uniformly distributed and structurally stable lithium silicate phases within the particle or at the interface. As a result, the amount of residual or loosely bound surface lithium is reduced, which suppresses side reactions with the electrolyte and improves the initial coulombic efficiency. Meanwhile, this also contributes to enhanced air and moisture stability. In summary, the CSALi-to-Si mass ratio and calcination temperature jointly regulate the formation of Li_2_Si_2_O_5_ and LiAlSiO_4_ phases and their spatial distribution on the silicon surface, while the carbon network provides additional electronic conductivity and mechanical buffering. These factors collectively determine the ICE, reversible capacity, and cycling stability of micron-sized silicon anodes. Notably, a higher surface Li content does not necessarily correspond to a higher ICE; only when lithium species are present in the form of stable lithium silicates and are uniformly distributed to form a continuous functional network can efficient pre-lithiation and superior electrochemical performance be achieved.

**Fig. 7:**
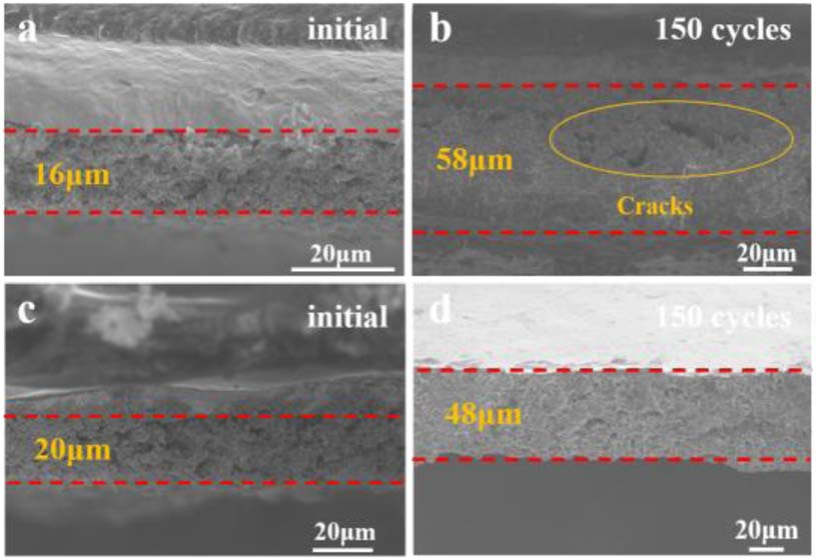
Cross-sectional SEM images of electrodes before and after 150 cycles at 1C: (a and b) pristine micron-sized silicon, (c and d) CLS12-1000 composite.

The cross-sectional SEM images after cycling (Fig. 7a–d) provide a clear comparison of structural stability between pristine micron-sized silicon and CLS12-1000 composite electrodes. For the pure silicon electrode (Fig. 7a and b), the initial thickness of 16 µm increases to 58 µm after 150 cycles at 1C, corresponding to a volume expansion of 262%, accompanied by pronounced through-thickness cracks and particle pulverization. This severe volumetric expansion and fragmentation indicate irreversible structural instability during repeated alloying/dealloying, leading to rapid capacity decay. In contrast, the CLS12-1000 electrode (Fig. 7c and d) shows a smaller thickness increase from 20 µm to 48 µm, corresponding to a volume expansion of 140%, with no observable through-thickness cracks, and the electrode structure remains dense and continuous. This improvement is primarily attributed to the *in situ* formed LiAlSiO_4_ phase. LiAlSiO_4_ establishes continuous Li^+^ conduction pathways at the interface, mitigating localized stress accumulation, while its negative thermal expansion helps buffer the volumetric changes of silicon, suppressing crack propagation. Furthermore, the spatially coupled network of LiAlSiO_4_, Li_2_Si_2_O_5_, and carbon effectively anchors the active silicon particles, maintaining electrode integrity. Therefore, compared with pure silicon, CLS12-1000 exhibits significantly reduced volume expansion (140% *vs.* 262%) and enhanced structural preservation after prolonged cycling, highlighting the critical role of LiAlSiO_4_ in mitigating volume changes, promoting uniform stress distribution, and improving cycling stability.

**Fig. 8:**
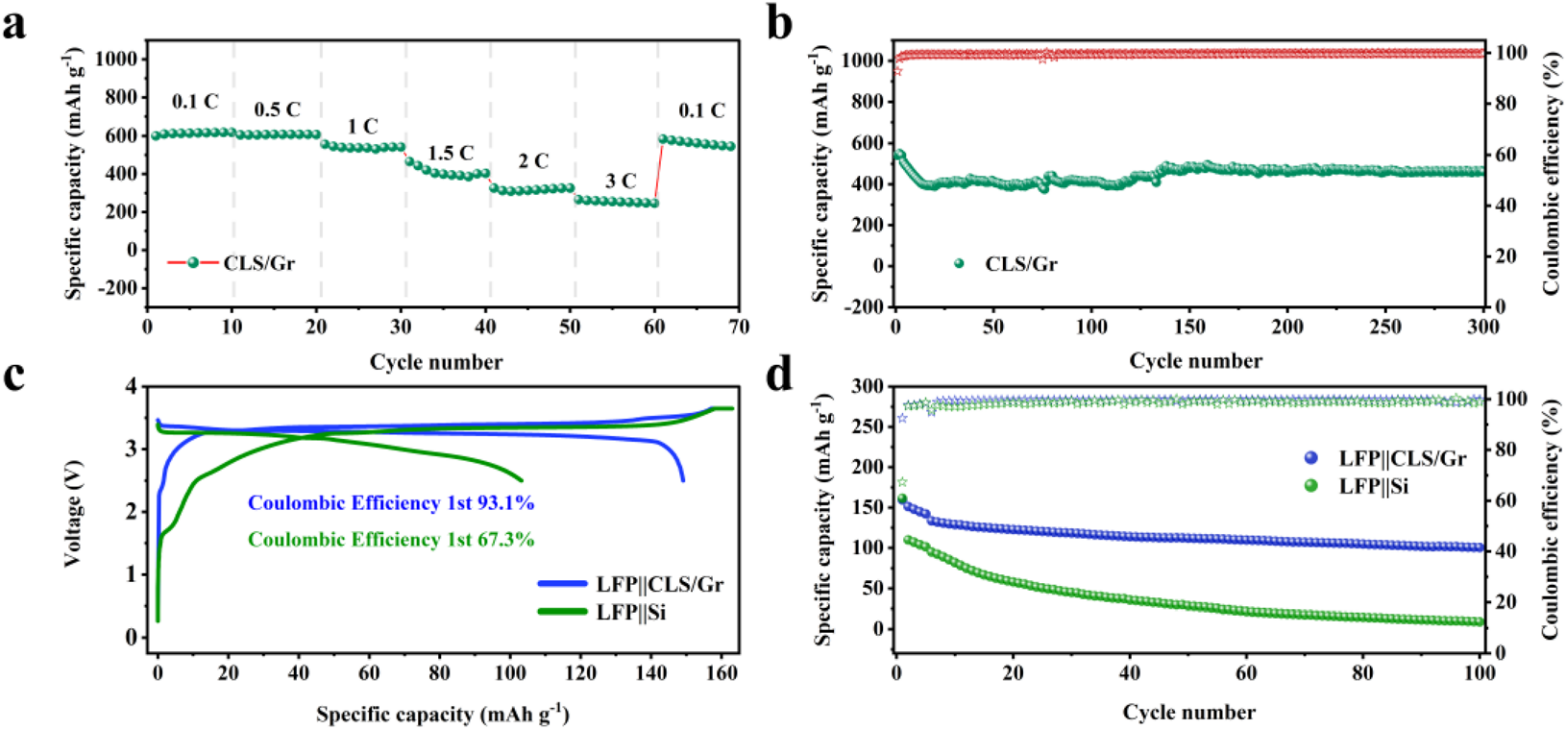
(a) Rate performance of CLS/Gr composite, (b) the cycling performance and the CE of CLS/Gr composite at 1C rate, (c) initial charge–discharge profiles of LFP‖CLS/Gr and LFP‖Si full cells at 0.1C, (d) long-term cycling performance of LFP‖CLS/Gr and LFP‖Si full cells at 0.5C.

The practical application potential of the composite was assessed by integrating CLS12-1000 with commercial graphite at a 1 : 4 mass ratio (CLS/Gr). As shown in the rate performance results in Fig. 8a, the CLS/Gr anode delivered a charge specific capacity of 600.6 mA h g^−1^ at 0.1C, retained a reversible capacity of 270.7 mA h g^−1^ when the rate increased to 3C, and recovered to 608.2 mA h g^−1^ when the rate returned to 0.1C. In the cycling stability test at 1C rate (Fig. 8b), the CLS/Gr anode still maintained a reversible specific capacity of 461.2 mA h g^−1^ after 300 cycles, with a capacity retention rate of 85.1%. Although the reversible specific capacity has slightly declined, considering the huge volume expansion of micron-silicon, this result still demonstrates the potential of the multi-component synergy strategy.

The full-cell performance was evaluated with the first five cycles conducted at 0.1C, followed by 100 cycles at 0.5C within a voltage window of 2.5–3.7 V, and a negative-to-positive capacity ratio (N/P ratio) of 1.1. As shown in Fig. 8c, the LFP‖CLS/Gr full cell delivered an initial discharge/charge capacity of 159.5/148.5 mA h g^−1^, corresponding to an initial coulombic efficiency (ICE) of 93.1% at 0.1C. In contrast, the LFP‖Si full cell exhibited a discharge/charge capacity of 163.0/109.7 mA h g^−1^, with a much lower ICE of 67.3%, indicating significant irreversible lithium loss in the pure Si negative electrode. After 100 cycles at 0.5C, the LFP‖CLS/Gr full cell retained a reversible capacity of 100.6 mA h g^−1^, corresponding to an energy density of approximately 332 W h kg^−1^ and a capacity retention of 67.7% (Fig. 8d). By comparison, the LFP‖Si cell retained only 9.1 mA h g^−1^, demonstrating poor cycling stability. The improved performance of CLS/Gr electrodes can be attributed to the synergistic effects of the Li_2_Si_2_O_5_/LiAlSiO_4_ functional phases and the carbon network, which effectively buffer the volume expansion of silicon, enhance interfacial lithium-ion transport, and maintain electronic connectivity. These results indicate that the CLS/Gr electrode not only provides high initial capacity and ICE but also maintains improved cycling stability in full cells. From a practical perspective, the present strategy is based on ball milling and high-temperature solid-state treatment, both of which are common powder-processing methods and are more suitable for scale-up than electrochemical or solution-based pre-lithiation methods involving highly reactive reagents. Moreover, the use of LiOH and fly ash-derived activated carbon avoids highly reactive lithium metal, SLMP, LiH, or organolithium reagents, improving the safety and cost advantages of the process. The improved full-cell ICE and cycling stability demonstrate that the multifunctional interface is beneficial not only in half cells but also in graphite-blended practical anode configurations.

**Fig. 9:**
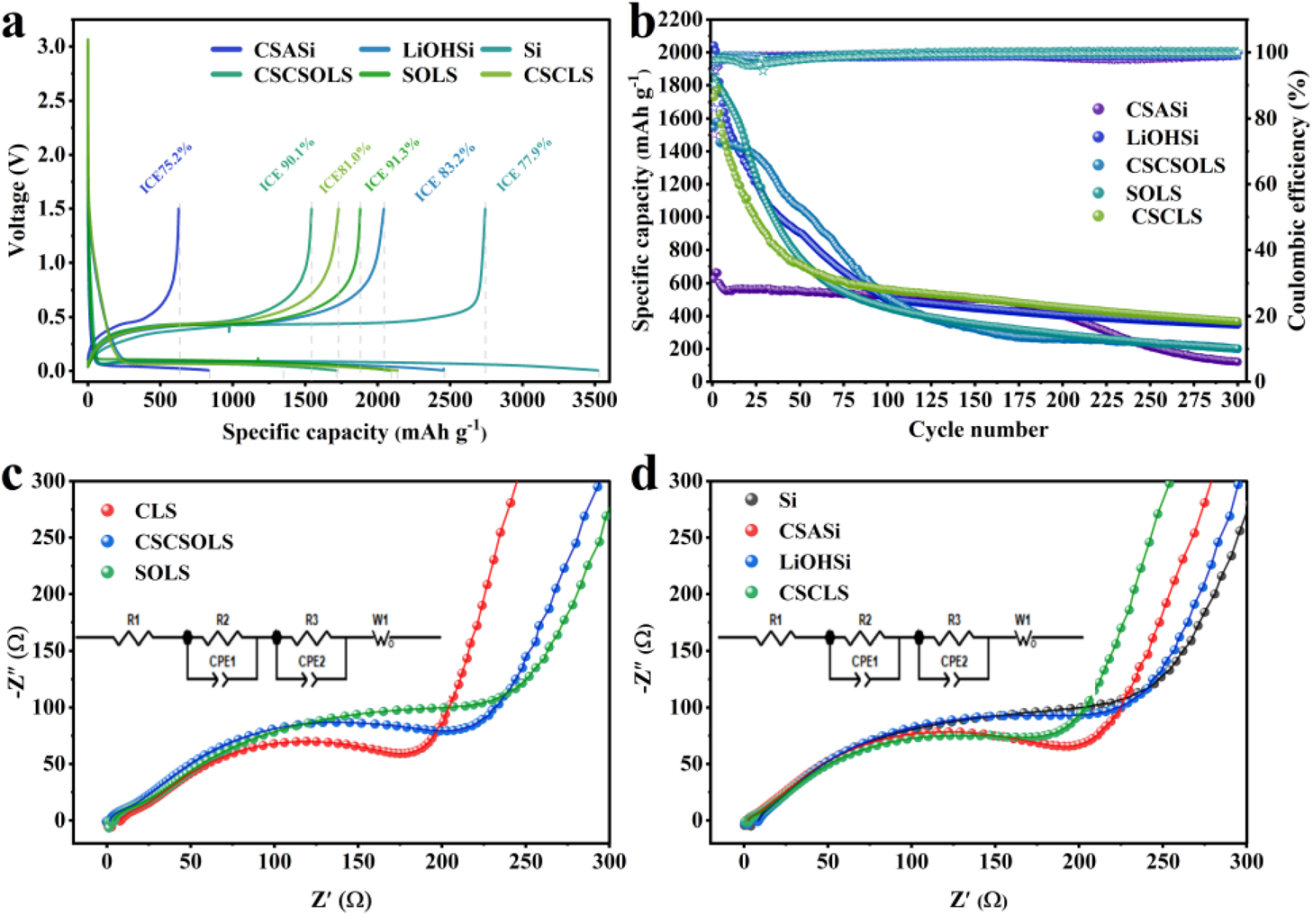
(a) Initial charge–discharge voltage profiles of comparative samples; (b) long-term cycling performance of comparative samples at 1C rate; (c) electrochemical impedance spectroscopy (EIS) spectra of Li_2_Si_2_O_5_-containing samples; (d) EIS spectra of comparative samples.

To investigate the contribution of individual functional components to the electrochemical performance of the micron-silicon anode, a series of comparative samples were designed and prepared. Table 1 summarizes the electrochemical performance of the main samples. Ball-milled micron-silicon (Si) exhibited a high discharge specific capacity of 3521.5 mA h g^−1^ but a low ICE of only 77.9% (Fig. 9a), with capacity dropping below 100 mA h g^−1^ after 20 cycles at 1C, indicating that mechanical ball milling alone cannot mitigate silicon volume expansion and irreversible lithium loss. The CSASi sample, containing only CSA and Si without lithium source addition, showed a low ICE of 75.2%, and its reversible capacity significantly decayed due to the presence of inert SiO_2_ and aluminosilicates. However, it exhibited relatively high cycling retention, attributed to the structural stabilization effect of irreversibly formed Li_*x*_SiO_*y*_ during electrochemical cycling.^33^ The LiOHSi sample, containing only LiOH and Si, achieved an ICE of 83.2%, indicating that lithium source addition can partially compensate for the irreversible capacity loss of the micron-silicon anode, but the pre-lithiation effect was limited due to the lack of stable pre-lithiation structures. When SiO_2_ and LiOH were pre-ball-milled to form Li_*x*_SiO_*y*_ (Fig. S6) before compositing with micron-silicon (SOLS), the ICE significantly improved to 91.31%, confirming that Li_*x*_SiO_*y*_ as pre-lithiation agents can effectively compensate for SEI related lithium loss. On this basis, the CSCSOLS sample (CSC + SiO_2_/LiOH + Si), with pure-phase coconut shell-derived carbon (CSC) as the carbon source, maintained a high ICE of 90.14% and achieved a 50-cycle capacity retention of 68.45% (Fig. 9b), significantly outperforming SOLS without CSC addition (40.02%), demonstrating that activated carbon can buffer the internal silicon volume expansion and enhance the cycling stability of anode materials. Meanwhile, both the ICE and 100-cycle capacity retention of the CSCSOLS sample were lower than those of the CLS sample, highlighting the critical role of the Li^+^-conducting phase LiAlSiO_4_. These results indicate that the performance enhancement of CLS cannot be attributed to any single component, but arises from the synergistic effect of Li_2_Si_2_O_5_, LiAlSiO_4_, and carbon within the spatially coupled interface. Pristine Si shows high initial capacity but low ICE and rapid capacity decay, reflecting the intrinsic instability of micron-Si. CSASi lacks an external lithium source and therefore cannot effectively compensate for initial lithium loss. LiOHSi contains LiOH but lacks the CSA-derived SiO_2_/Al_*x*_SiO_*y*_ framework required for constructing the complete multifunctional interface. SOLS confirms that lithium silicate species can improve ICE, but its cycling stability remains limited. CSCSOLS shows improved cycling stability after carbon introduction, demonstrating the buffering and conductive role of carbon. However, its overall performance is still inferior to CLS12-1000, indicating the additional contribution of the LiAlSiO_4_-containing interface. Therefore, the optimized performance of CLS12-1000 originates from the spatial coupling of lithium compensation, interfacial Li^+^ transport, electronic conduction, and structural buffering.

**Table 1:** Electrochemical performance of samples

Sample	Discharge (mA h g^−1^)	Charge (mA h g^−1^)	ICE (%)	Capacity retention (50 cycles)/%	Capacity retention (100 cycles)/%
CLS12-1000	1676.5	1564.3	93.3	70.72	50.43
Si	3521.5	2742.6	77.9	0.13	0.095
CSASi	833.1	626.7	75.2	83.55	79.32
LiOHSi	2455.9	2042.6	83.2	44.28	24.46
SOLS	2082.1	1880.3	91.3	40.02	23.86
CSCSOLS	1713.1	1544.2	90.1	68.45	33.23
CSCLS	2136.9	1730.6	81.0	41.09	32.37

It is noteworthy that the capacity difference among different samples gradually diminishes after prolonged cycling. This phenomenon indicates that the multifunctional interface mainly plays a dominant role during the initial and early-to-middle cycling stages by compensating irreversible lithium loss, reducing interfacial polarization, and buffering volume changes. During long-term cycling, however, micron-Si still inevitably undergoes repeated volume expansion/contraction, particle pulverization, continuous SEI evolution, and partial loss of electrical contact. These common degradation processes gradually reduce the amount of electrochemically active Si in different electrodes, leading to the convergence of the remaining reversible capacities. Therefore, the Li_2_Si_2_O_5_/LiAlSiO_4_/C network can significantly delay, but cannot completely eliminate, the intrinsic degradation of micron-Si.

Electrochemical impedance spectroscopy (EIS) tests were conducted on the composite material and the control sample before the half-cell cycling. In the equivalent circuit model, *R*_1_, *R*_2_, and *R*_3_ respectively represent the electrolyte, the SEI film, and the charge transfer resistance.^57^ The Nyquist plots of the composite samples containing Li_*x*_SiO_*y*_ lithium-adding agents show a small semi-circle in the high-frequency region and a larger semi-circle in the medium-frequency region (Fig. 9c), while the samples without the addition of Li_*x*_SiO_*y*_ only show a single semi-circle (Fig. 9d). This is attributed to the presence of Li_*x*_SiO_*y*_ phase in the composite sample. In the presence of the electrolyte, an SEI film was pre-formed, resulting in the SEI film resistance.^58^ Based on the comparison of the interface resistances *R* = (*R*_2_ + *R*_3_) of each material after fitting, it is shown that the interface resistance of the CLS composite material (184.8 Ω) is lower than that of CSCSOLS (226.5 Ω), SOLS (251.1 Ω), Si (240.1 Ω), CSASi (207.5 Ω), LiOHSi (231.2 Ω), and CSCLS (205.5 Ω). This is attributed to the synergistic effect between the conductive network provided by the carbon material in the composite material and the *in situ* generated LiAlSiO_4_ fast ionic conductor.

## 4. Conclusions

In summary, a facile *in situ* solid-state reaction strategy was developed to construct a multifunctional Li_2_Si_2_O_5_/LiAlSiO_4_/C network on micron-Si anodes. Using fly ash-derived activated carbon as a multifunctional precursor and LiOH as the lithium source, Li_2_Si_2_O_5_, LiAlSiO_4_, and carbon were spatially coupled around Si particles through ball milling and high-temperature treatment. In this integrated interface, Li_2_Si_2_O_5_ provides lithium compensation, LiAlSiO_4_ facilitates interfacial Li^+^ transport, and the carbon network improves electronic conductivity and buffers volume variation. Benefiting from this multifunctional interface, the optimized CLS12-1000 composite delivered initial discharge/charge capacities of 1676.5/1564.3 mA h g^−1^ with a high ICE of 93.3%, significantly higher than that of pristine micron-Si (77.9%). The interface also reduced the electrode expansion after cycling from 262% for pristine Si to 140% for CLS12-1000, confirming improved structural stability. When blended with commercial graphite, the CLS/Gr electrode retained 461.2 mA h g^−1^ after 300 cycles at 1C with a capacity retention of 85.1%. Full-cell tests further demonstrated improved practical performance, with the LFP‖CLS/Gr cell delivering an ICE of 93.1%, much higher than that of the LFP‖Si cell (67.3%), and retaining 100.6 mA h g^−1^ after 100 cycles at 0.5C. These results demonstrate that the enhanced performance originates from the synergistic effects of lithium compensation, interfacial ion transport, electronic conduction, and structural buffering. This work provides a low-cost and scalable route for the high-value utilization of fly ash-derived carbon and offers a promising design strategy for high-ICE micron-Si-based anodes.

## Author contributions

Jingyu Yang: data curation, formal analysis, investigation, methodology, software, visualization, writing – original draft. Xikai Zhou: project administration, resources, validation. Junfeng Rong: conceptualization, funding acquisition, supervision, writing – review and editing.

## Conflicts of interest

There are no conflicts to declare.

## Supplementary Material

RA-016-D6RA02190E-s001

## Data Availability

All data related to this study are available from the corresponding author. Supplementary information (SI): additional material characterization results. See DOI: https://doi.org/10.1039/d6ra02190e.
