# Supplementary material for: In situ construction of multifunctional Li2Si2O5/LiAlSiO4/C networks on micron silicon anodes for high initial coulombic efficiency
Source: RSC Adv. 2026 May 26;16(31):28280–91. doi: 10.1039/d6ra02190e (PMC13213564; doi:10.1039/d6ra02190e)
Supplement: RA-016-D6RA02190E-s001 [file RA-016-D6RA02190E-s001.pdf]

Supplementary Information (SI) for RSC Advances.
This journal is © The Royal Society of Chemistry 2026

In-situ Construction of Multifunctional $Li_2Si_2O_5/LiAlSiO_4/C$ Networks on Micron Silicon Anodes for High Initial Coulombic Efficiency

Jingyu Yang, Xikai Zhou and Junfeng Rong*

Sinopec Research Institute of Petroleum Processing Co., Ltd. Beijing, 100083, PR China

* Corresponding author: Rongjunfeng.ripp@sinopec.com

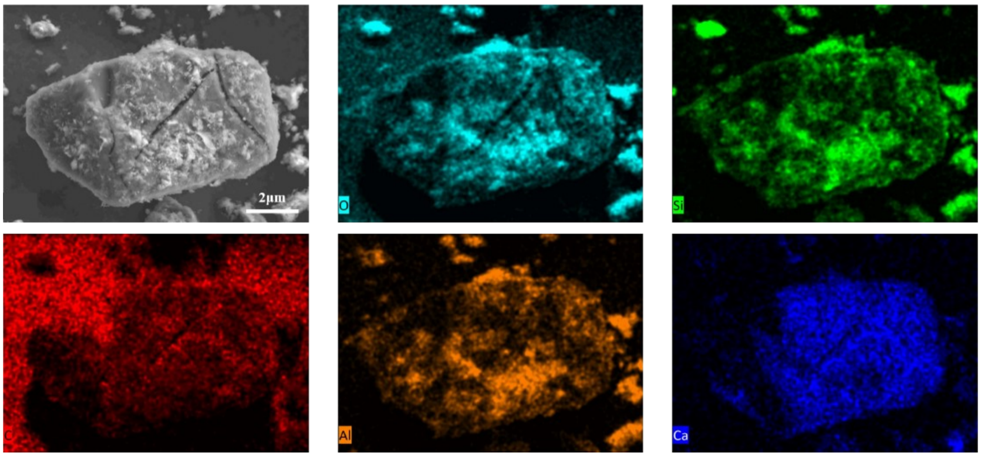

Fig.S1:EDS mapping of CSA.

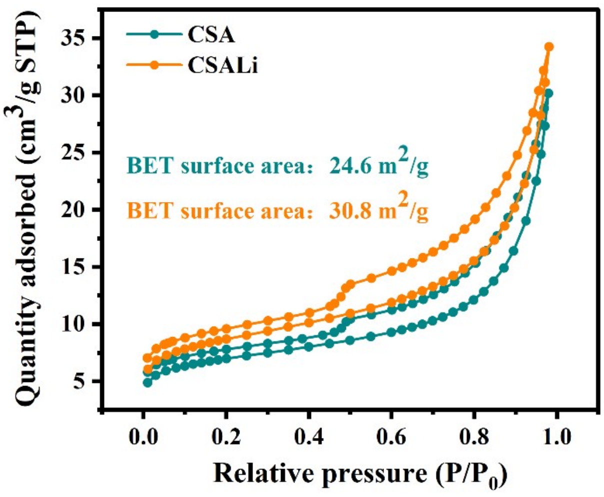

Fig.S2: BET of CSA and CSALi.

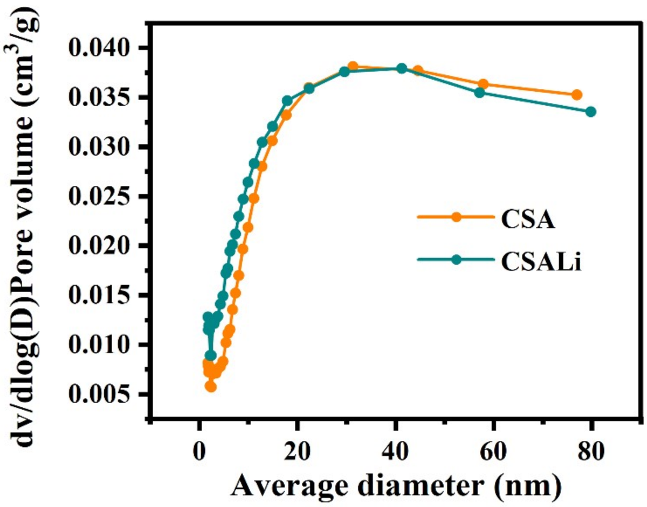

Fig.S3: Pore volume of CSA and CSALi.

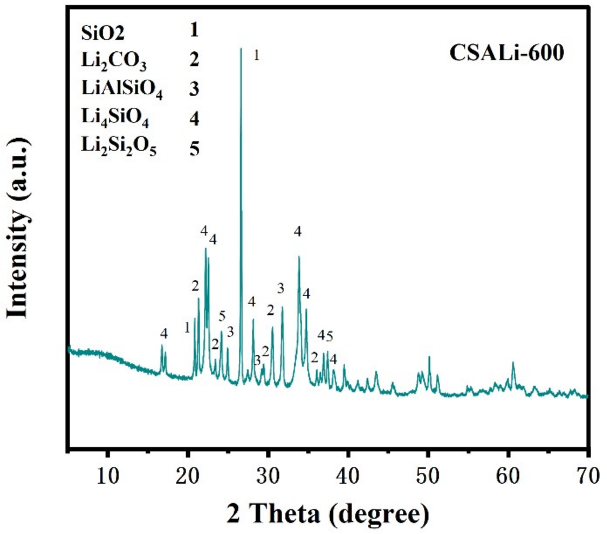

Fig.S4: XRD of CSALi (Excessive Li).

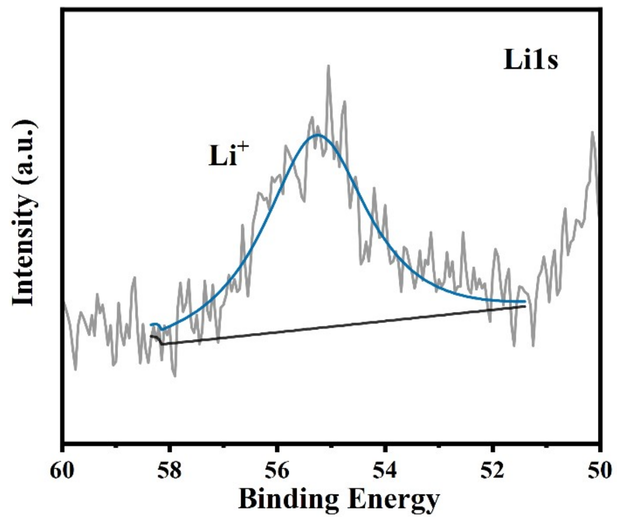

Fig.S5: Li1s of CLS12-1000.

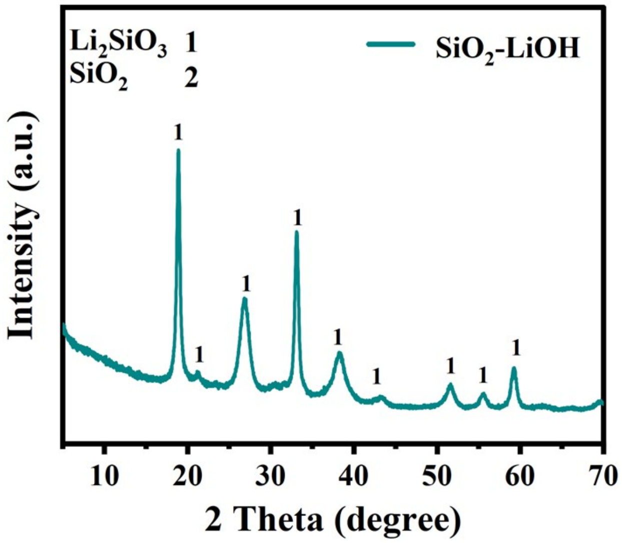

Fig.S6: XRD of pre-ball-milling SiO_2 and LiOH sample.
